# Novel Antifungal Compounds Discovered in Medicines for Malaria Venture’s Malaria Box

**DOI:** 10.1128/mSphere.00537-17

**Published:** 2018-03-14

**Authors:** Eric H. Jung, David J. Meyers, Jürgen Bosch, Arturo Casadevall

**Affiliations:** a Department of Microbiology and Immunology, Albert Einstein College of Medicine, Bronx, New York, USA; b Department of Molecular Microbiology and Immunology, Johns Hopkins Bloomberg School of Public Health, Baltimore, Maryland, USA; c Department of Biochemistry and Molecular Biology, Johns Hopkins Bloomberg School of Public Health, Baltimore, Maryland, USA; d Division of Pulmonology and Allergy/Immunology, Case Western Reserve University, Cleveland, Ohio, USA; e Department of Pharmacology and Molecular Sciences, Johns Hopkins School of Medicine, Baltimore, Maryland, USA; f InterRayBio, LLC, Baltimore, Maryland, USA; Carnegie Mellon University

**Keywords:** *Candida albicans*, *Cryptococcus neoformans*, antifungal agents

## Abstract

Similarities in fungal and animal cells make antifungal discovery efforts more difficult than those for other classes of antimicrobial drugs. Currently, there are only three major classes of antifungal drugs used for the treatment of systemic fungal diseases: polyenes, azoles, and echinocandins. Even in situations where the offending fungal organism is susceptible to the available drugs, treatment courses can be lengthy and unsatisfactory, since eradication of infection is often very difficult, especially in individuals with impaired immunity. Consequently, there is a need for new and more effective antifungal drugs. We have identified compounds with significant antifungal activity in the Malaria Box (Medicines for Malaria Ventures, Geneva, Switzerland) that have higher efficacy than some of the currently used antifungal drugs. Our best candidate, MMV665943 (IUPAC name 4-[6-[[2-(4-aminophenyl)-3H-benzimidazol-5-yl]methyl]-1H-benzimidazol-2-yl]aniline), here referred to as DM262, showed 16- to 32-fold-higher activity than fluconazole against *Cryptococcus neoformans*. There was also significant antifungal activity in other fungal species with known antifungal resistance, such as *Lomentospora prolificans* and *Cryptococcus gattii*. Antifungal activity was also observed against a common fungus, *Candida albicans*. These results are important because they offer a potentially new class of antifungal drugs and the repurposing of currently available therapeutics.

**IMPORTANCE** Much like the recent increase in drug-resistant bacteria, there is a rise in antifungal-resistant strains of pathogenic fungi. There is a need for novel and more potent antifungal therapeutics. Consequently, we investigated a mixed library of drug-like and probe-like compounds with activity in *Plasmodium* spp. for activity against two common fungal pathogens, *Cryptococcus neoformans* and *Candida albicans*, along with two less common pathogenic species, *Lomentospora prolificans* and *Cryptococcus gattii*. We uncover a previously uncharacterized drug with higher broad-spectrum antifungal activity than some current treatments. Our findings may eventually lead to a compound added to the arsenal of antifungal therapeutics.

## Abstract (precis)

Much like the recent increase in drug-resistant bacteria, there is a rise in antifungal-resistant strains of pathogenic fungi. There is a need for novel and more potent antifungal therapeutics. Consequently, we investigated a mixed library of drug-like and probe-like compounds with activity in *Plasmodium* spp. for activity against two common fungal pathogens, *Cryptococcus neoformans* and *Candida albicans*, along with two less common pathogenic species, *Lomentospora prolificans* and *Cryptococcus gattii*. We uncover a previously uncharacterized drug with higher broad-spectrum antifungal activity than some current treatments. Our findings may eventually lead to a compound added to the arsenal of antifungal therapeutics.

## INTRODUCTION

The global burden of fungal diseases is a major human problem, especially in developing and rural regions where diagnostics and treatment are difficult to distribute. The access to drugs currently used to treat potentially life-threatening fungal diseases is inefficient and costly. A study in 2002 projected an average incidence of fungal infection at $306 per million U.S. population, estimating a total direct cost of $2.6 billion and an average of $31,200 per patient (1). When we transpose these data onto the areas of the highest-affected population in sub-Saharan Africa, where the average annual income per capita is approximately $750, the cost for treatment is generally unattainable (2).

Candidiasis and cryptococcosis are two widespread human mycoses, caused by ascomycetous *Candida* spp. and basidiomycetous *Cryptococcus* spp., respectively. *Candida* spp. are found in the commensal flora and can cause a diverse set of diseases in humans ranging from mucosal to systemic infection, the most common species being *Candida albicans*. While the primary niche of *Candida* spp. is the gastrointestinal tract, the organism can cause disease in various body sites, producing oropharyngeal and vaginal candidiasis (3). With a breach in mucosal lining or immunosuppression, local candidiasis can transform into systemic disease that can disseminate, causing candidemia, meningitis, or deep organ disease with high fungal burden (4). *Cryptococcus neoformans* is a facultative intracellular pathogen found primarily among HIV/AIDS-infected individuals, resulting in 180,000 deaths per annum, predominantly in sub-Saharan Africa (5). Infection occurs via inhalation of spores or desiccated yeast cells and is controlled by alveolar macrophages phagocytosing the pathogen. Cryptococcosis is comprised of pneumonia and meningoencephalitis, acute swelling of the brain and meninges, and cryptococcomas, small tumor-like masses of infection, both of which can subsequently lead to an intracranial buildup in pressure (6). *Cryptococcus gattii* is a related species but, however, far rarer, as only 218 cases were reported in British Columbia, Canada, during 1999 to 2007 (7) and 96 cases were reported in the Pacific Northwest of the United States during 2004 to 2011 (8). While *C. neoformans* normally affects immunocompromised individuals, the hallmark characteristic of *C. gattii* is the ability to cause disease in healthy, immunocompetent individuals (9). *C. gattii* is also known for natural resistance to the typical azoles used in the treatment of cryptococcosis (10, 11).

*Lomentospora prolificans*, formerly *Scedosporium prolificans*, is found worldwide and is naturally highly resistant to existing antifungal drugs. *L. prolificans* is an emerging pathogen that causes fatal infection in immunocompromised hosts (12). This organism can also cause disease in immunocompetent hosts in the form of debilitating skin, soft tissue, and bone (mycetoma) infections (13). A notable characteristic of *L. prolificans* is the intrinsic antifungal resistance to common antifungal drugs such as amphotericin B, flucytosine, fluconazole, itraconazole, ketoconazole, miconazole, and voriconazole (14). Clinical manifestation of *L. prolificans* can begin as a localized lesion from trauma or inhalation which can develop into systemic infection due to its capacity to produce conidia in bodily fluids (12). Current treatment for *L. prolificans* infection is surgical debridement of affected tissue along with systemic high-dose antifungal therapy (15).

In 2010, Gamo et al. at GlaxoSmithKline (GSK) screened approximately 2 million compounds for antimalarial leads. They discovered 13,533 compounds that inhibited growth by more than 80% at a 2 μM concentration in *Plasmodium falciparum*, the *Plasmodium* species associated with the highest malaria-related mortality (16). A subset of these compounds was made available to interested users in the form of the Medicines for Malaria Venture (MMV) Malaria Box (17). The compounds were selected to be chemically diverse and cover most scaffolds that inhibited growth of *Plasmodium* parasites. Since the distribution of these compounds, multiple research laboratories have performed drug screening on various human pathogens (18). In this study, we screened the open-source MMV Malaria Box compound library for potentially novel antifungal compounds.

## RESULTS

### Primary screen and dose-response of selected candidates.

**FIG 1:**
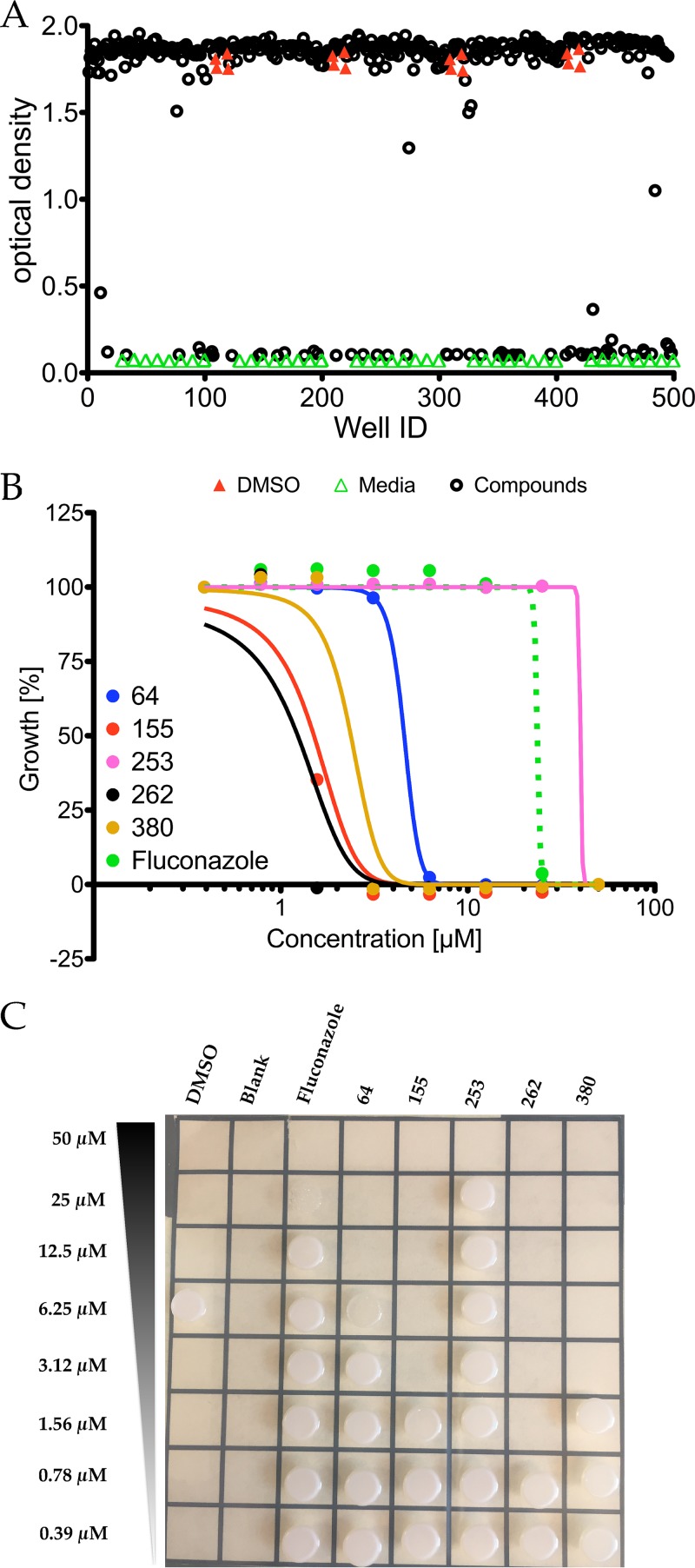
Primary screening results of the MMV Malaria Box library. (A) The 400 Malaria Box compounds were screened at a 50 µM concentration in 0.5% DMSO for activity against *C. neoformans* in liquid cultures. DMSO vehicle controls are indicated with red closed triangles, medium control without fungal growth is indicated with green open triangles, and wells containing drug are indicated with black open circles. (B) Dose-response curve of selected compounds. *C. neoformans* cultures were plated and exposed to DMSO vehicle control or inhibitors for 72 h ranging from 0.39 µM to 50 µM concentrations. A parent molecule, PTA-D0 (26, 27), of our 240-derivative library was included in the initial screen; however, it did not prove fungicidal. (C) After treatment, the liquid cultures were plated on Sabouraud rich medium and grown at 30°C to allow full recovery.

**FIG 2:**
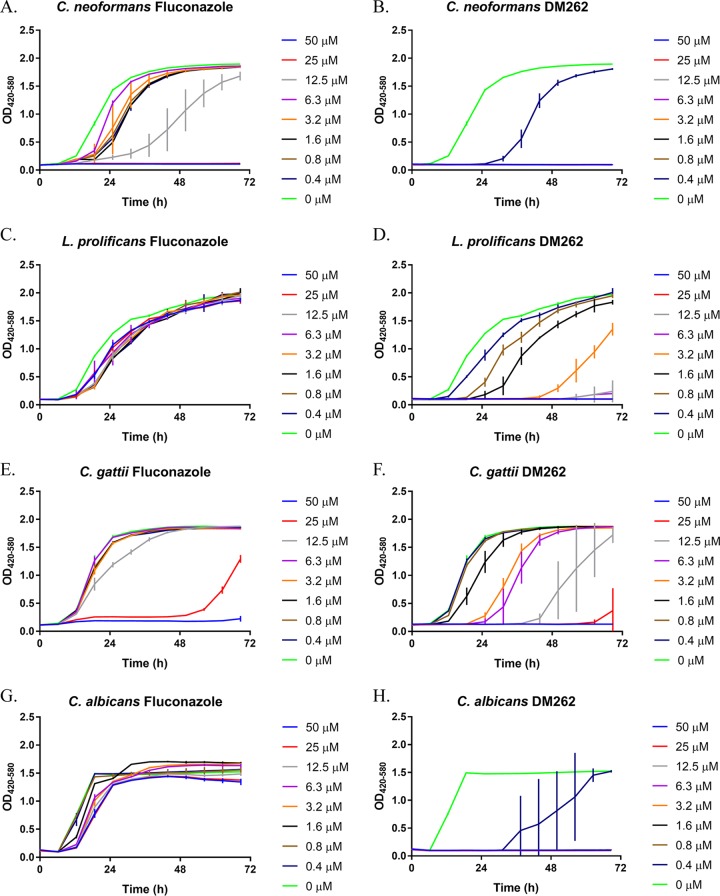
Dose-dependent growth inhibition assays of fluconazole and DM262. A 2-fold dilution series of fluconazole and DM262 in DMSO treatment in a variety of pathogenic fungal species. OD was measured in wideband (420 to 580 nm) to measure turbidity while accounting for differences in medium color. The difference in growth inhibition between fluconazole and DM262 is shown in *Cryptococcus neoformans* (A and B), *Lomentospora prolificans* (C and D), *Cryptococcus gattii* (E and F), and *Candida albicans* (G and H). Error bars represent standard deviations within technical triplicates. Each experiment had at least two biological replicates.

To identify antifungal candidates in the MMV Malaria Box, we initially cast the widest possible net. *C. neoformans* growth inhibition assays in the presence of the 400 antimalarial compounds were done by following fungal growth as reflected by increases in turbidity using the Bioscreen C instrument set with constant agitation in rich medium and at a physiologically relevant temperature of 37°C to test the 400 antimalarial compounds against *C. neoformans*. We identified 56 compounds that inhibited *C. neoformans* (Fig. 1A) at a 50 µM final concentration in our primary screen. To narrow down our candidate compounds, we repeated growth inhibition assays on our 56 candidates in 2-fold dilution series for their dose-dependent activity. Five were selected based on their fungicidal activity relative to fluconazole, which is a commonly used antifungal drug against *C. neoformans* (Table 1; Fig. 1B). To examine whether or not each dose was fungicidal or fungistatic, we plated a sample of each dilution after 72 h. Cells were plated on Sabouraud (Sab) agar and allowed to grow for 24 h, and colonies were enumerated (Fig. 1C). The most promising compound was MMV665943 (IUPAC name 4-[6-[[2-(4-aminophenyl)-3H-benzimidazol-5-yl]methyl]-1H-benzimidazol-2-yl]aniline), here referred to as DM262. DM262 demonstrated fungicidal activity down to 1.56 μM and fungistatic activity down to 0.8 μM, concentrations that are 16- and 32-fold more effective, respectively, than fluconazole against *C. neoformans* (Fig. 2A and B). We assigned our own compound numbers to more easily keep track of all compounds screened. Compounds MMV665807 (which we called compound 155) and MMV665882 (compound 380) were similarly effective, showing fungicidal activity at 3.12 μM, still 8-fold more effective than fluconazole. MMV007374 (compound 64) and MMV666079 (compound 253) were also assessed for fungicidal activity, but their low activity as well as the low activities of compounds 155 and 380 dissuaded us from further pursuit (Fig. 1B and C). To assess the fungicidal activity in other species, we needed to synthesize more MMV665943, further referred to as DM262 (see Fig. S2 in the supplemental material).

10.1128/mSphere.00537-17.1 FIG S1  Emission spectrum. To assess the emission spectrum for DM262 and DMSO vehicle control, we identified 391 to 393 nm as the optimal wavelength for emission. We excited both DM262 and DMSO vehicle control at 400 nm and concluded that it was indeed possible to visualize this compound via fluorescence microscopy. Download FIG S1, PDF file, 0.1 MB. Copyright © 2018 Jung et al. 2018 Jung et al. This content is distributed under the terms of the Creative Commons Attribution 4.0 International license.

10.1128/mSphere.00537-17.2 FIG S2  4,4′-Methylenebis(2-nitroaniline) (compound 1) was synthesized according to the work of Chauvin et al. (Chem Eur J 13:9515–9526, 2007, https://doi.org/10.1002/chem.200700883); we obtained an orange solid in 92% crude yield which contained ~5 to 10% 2-nitroaniline. Due to the compound’s poor solubility in several organic solvents, the material was used in the next step without further purification. ^1^H nuclear magnetic resonance (NMR) data: (500 MHz, DMSO-d6) d 7.81 (d, *J* = 1.73 Hz, 2H), 7.35 (s, 4H), 7.27 (dd, *J* = 2.04, 8.80 Hz, 2H), 6.96 (d, *J* = 8.65 Hz, 2H), 3.75 (s, 2H). Liquid chromatography-mass spectrometry (LC-MS): retention time (Rt) = 2.43 min; *m/z* = 289.1 [M + H]^+^. The syntheses of compounds 2 and 3 were adapted from the procedure described for 6,10-dihydrofluoreno[2,3-*d*:6,7-*d*′]diimidazole derivatives (H.-F. Chen, Y.-M. Cui, J.-G. Guo, and H.-X. Lin, Dyes Pigm 94:583–591, 2012, https://doi.org/10.1016/j.dyepig.2012.03.004). 4,4′-Methylenebis(benzene-1,2-diamine) (compound 2) was synthesized as follows. To an orange suspension of 4,4′-methylenebis(2-nitroaniline) (compound 1) (533 mg, 1.85 mmol) in ethanol (EtOH) (37 ml) was added Pd(OH)_2_ (106 mg; 20 wt% Pd on wet carbon). The reaction mixture was heated to reflux, and hydrazine hydrate was added dropwise until starting material was consumed. The reaction mixture was removed from heat and allowed to cool to room temperature (RT). The Pd(OH)_2_/C was removed by filtration, and the EtOH was removed *in vacuo*. The residue was used in the next step without further purification. LC-MS: Rt = 0.49 to 0.51 min; *m/z* = 229.2 [M + H]^+^. Bis(2-(4-nitrophenyl)-1H-benzo[*d*]imidazol-6-yl)methane (compound 3) was synthesized as follows. To crude compound 2 (~1.85 mmol) in anhydrous 1,4-dioxane (50 ml) was added 4-nitrobenzaldehyde (587 mg, 3.88 mmol). The reaction mixture was heated to reflux for 18 h under an argon atmosphere. After cooling to RT, the reaction mixture was absorbed onto silica gel and the volatiles were removed *in vacuo*. The reaction mixture was purified on silica gel using a hexane/ethyl acetate gradient. The fractions containing the desired product were pooled and purified on a flash C_18_ column using a water/acetonitrile gradient to provide compound 3 (51 mg, 6% yield) as a bright yellow solid. ^1^H NMR: (500 MHz, DMSO-d6) d 8.31 to 8.51 (m, 8H), 7.57 to 7.67 (m, *J* = 8.33 Hz, 2H), 7.55 (s, 2H), 7.16 to 7.30 (m, *J* = 8.49 Hz, 2H), 4.24 (s, 2H). LC-MS: Rt = 2.08 min; *m/z* = 491.0 [M + H]^+^. 4,4′-(6,6′-Methylenebis(1H-benzo[*d*]imidazole-6,2-diyl))dianiline (compound 4) was synthesized as follows. To an orange suspension of compound 3 (50 mg, 0.10 mmol) in EtOH (5 ml) was added Pd(OH)_2_ (15 mg; 20 wt% Pd on wet carbon). The reaction mixture was heated to reflux, and hydrazine hydrate was added dropwise until starting material was consumed. The reaction mixture was removed from heat and allowed to cool to RT. The Pd(OH)_2_/C was removed by filtration, and the EtOH was removed *in vacuo*. The remaining solid was purified on a flash C_18_ column using a water/acetonitrile gradient to provide compound 4 (24 mg, 55% yield) as a tan solid. ^1^H NMR: (500 MHz, methanol-d4) d 7.80 (d, *J* = 8.49 Hz, 4H), 7.41 to 7.50 (m, *J* = 8.17 Hz, 2H), 7.37 (s, 2H), 7.07 to 7.17 (m, *J* = 8.02 Hz, 2H), 6.78 (d, *J* = 8.49 Hz, 4H), 4.20 (s, 2H). LC-MS: Rt = 1.70 min; *m/z* = 431.2 [M + H]^+^. Download FIG S2, PDF file, 0.1 MB. Copyright © 2018 Jung et al. 2018 Jung et al. This content is distributed under the terms of the Creative Commons Attribution 4.0 International license.

**TABLE 1:**
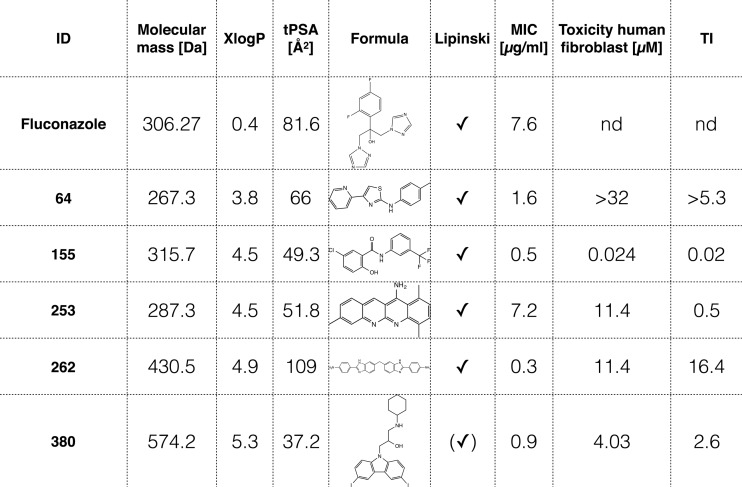
Identified and validated antifungal compounds from the MMV Malaria Box library with fungicidal activity comparable to or better than that of fluconazole^a^

a XlogP values are derived from PubChem and describe the lipophilicity of the molecule. The topological polar surface area (tPSA) is a metric for predicting cell penetration, with values of <140 being favorable. The MIC was determined after 72-h drug exposure in liquid culture and continued growth on yeast extract-peptone-dextrose agar plates overnight for recovery of the cultures. Toxicity against human fibroblasts was used to calculate the therapeutic index (TI). ID, identifier; Lipinski, Lipinski's rule of 5 to determine druglikeness of candidate compounds for oral bioavailability in humans; nd, not determined.

### DM262 activity against other fungal species.

We investigated the antifungal activity of DM262 against other fungal pathogens such as *Lomentospora prolificans* (Fig. 2C and D), *Cryptococcus gattii* (Fig. 2E and F), and *Candida albicans* (Fig. 2G and H). Growth inhibition assays with a 2-fold dilution series of DM262 in *L. prolificans* showed complete resistance to fluconazole even at the highest concentration tested, 50 μM. DM262 treatment was fungistatic at a concentration of 50 to 12.5 μM, and growth was delayed at concentrations of 3.2 μM to 0.39 μM. *Cryptococcus gattii* in the same assay showed resistance to fluconazole as growth was unaffected at 6.3 μM and lower. At 12.5 μM and higher concentrations of DM262, there was significant delay in the growth of *C. gattii*. Finally, when examining the susceptibility in *Candida albicans*, fluconazole yielded only a slight decrease in final optical density at 420 to 580 nm (OD_420−580_). The MICs have been compared for all the fungal pathogens studied here for fluconazole and DM262 (Table 2). The *L. prolificans* and *C. albicans* strains studied were classified as resistant to fluconazole as there was neither a fungicidal nor a fungistatic concentration up to 50 μM, the highest concentration that we tested (Table 2).

**TABLE 2:** MIC results

Species	MIC (μM)^a^	
	Fluconazole	DM262
*C. neoformans*	25	0.8
*L. prolificans*	Res	6.3
*C. gattii*	50	50
*C. albicans*	Res	0.8

a We measured the MIC after 72 h of fluconazole versus DM262 in four different fungal species: *C. neoformans*, *L. prolificans*, *C. gattii*, and *C. albicans*. “Res” denotes resistance to the highest concentration of drug that we analyzed, 50 μM.

### Mammalian cell toxicity.

**FIG 3:**
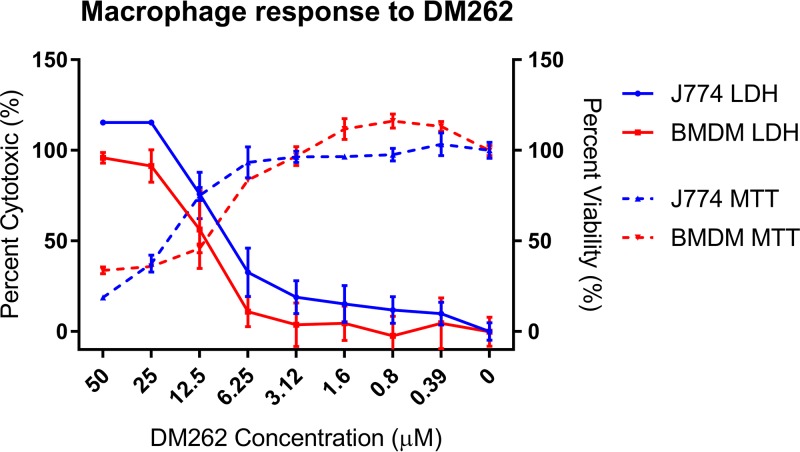
Mammalian cell cytotoxicity shown by macrophage response to DM262. Viability was measured in BMDM and J774 cells using an MTT assay on DM262 which is shown to have an LD_50_ of 12.5 μM. Cell lysis was measured by LDH release into the supernatant of BMDM, showing similar numbers with an LD_50_ of 12.5 μM. Data points were determined in triplicate, and this experiment was repeated twice. Shown are mean and standard deviation for each value.

To investigate the effects of DM262 on host mammalian cells, we examined cytotoxic lysis and viability in murine bone marrow-derived macrophages (BMDM) by measuring lactate dehydrogenase (LDH) and metabolic activity {[3-(4,5-dimethyl-2-thiazolyl)-2,5-diphenyl-2H-tetrazolium bromide] [MTT]}. The two assays yielded similar 50% lethal dose (LD_50_) values of 12.5 μM for DM262 and with minimal variance from dimethyl sulfoxide (DMSO) vehicle control up to 6.25 μM (Fig. 3).

### Internalization of DM262.

**FIG 4:**
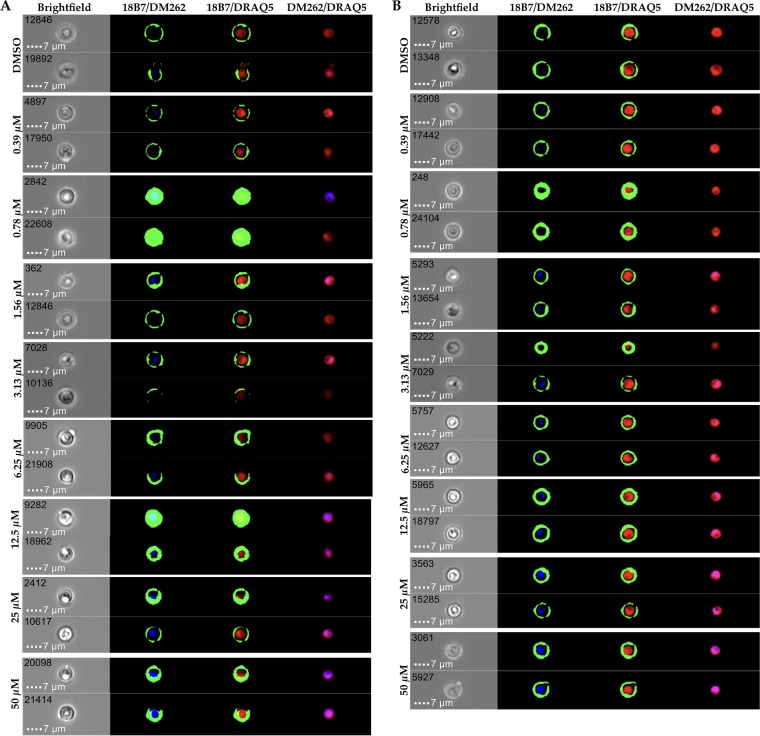
Internalization of DM262 in *C. neoformans*. Each of the panels shows the bright-field image at ×60 magnification and three overlaid images indicated by the respective channels. (A) Two populations of cryptococcal cells were treated with either DMSO or 0.39 µM to 50 μM DM262 for 24 h prior to fixing and analysis on an Amnis ImageStream MK II flow cytometer. DRAQ5-stained nuclei (red) and AF488 (green)-stained 18B7 conjugated to the polysaccharide capsule of *C. neoformans* outlining the exterior of the cell. DM262 appears to associate partially with the nuclei in our investigation. Two cells from each sample at a particular DM262 concentration were chosen as representatives of the population with the brightest DM262 fluorescence. A complete analysis of all concentrations is available as Fig. S3 in the supplemental material. Representative images from ImageStream analysis highlighting the main differences between untreated and treated cells are in Fig. S4. (B) Samples from panel A were further analyzed by binning the average intensity of DM262 for the respective sample. Two cells were randomly picked from the bin corresponding to the mean intensity of DM262 for each concentration. This bin typically contained more than 50 cells.

**FIG 5:**
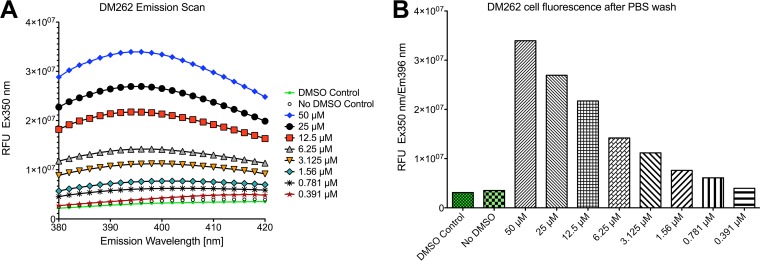
Change in fluorescence of *C. neoformans* upon addition of DM262. (A) Emission scan of *C. neoformans* cells exposed to DM262 at various concentrations. (B) Dose-dependent plot at optimal excitation and emission wavelength for DM262. Cells were previously washed twice in PBS to remove any residual DM262 in the medium. Fluorescence of control cells with and without DMSO is shown in the green bars. RFU, relative fluorescence units.

**FIG 6:**
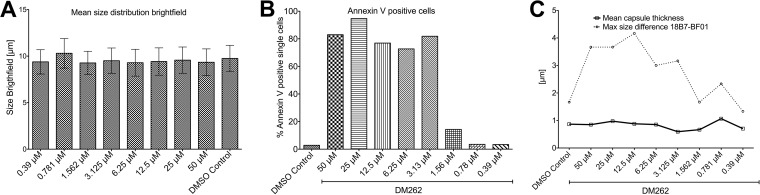
Morphological characteristics of DM262-treated *C. neoformans* cells. (A) The mean size distribution of DM262-treated *C. neoformans* samples was analyzed using the bright-field information acquired on the ImageStream flow cytometer. Each sample contained >10,000 single cells. No meaningful change in size is apparent between treated and control samples. (B) Cells staining positive for annexin V were used as a proxy for apoptosis-like cell death of *C. neoformans* DM262-exposed cells. Representative plots for the annexin V gating strategy are available as Fig. S5 in the supplemental material. Plots for all concentrations are represented as Fig. S6. (C) Evaluation of the thickness of the polysaccharide capsule by ImageStream analysis. The mean thickness calculated between the diameter difference of the 18B7 image and the bright-field image is shown as a solid line. The largest cell radius differences occurring between 18B7 staining and bright-field images are shown as a dotted line. A DM262 dose-dependent shift in size is observed. The graph in panel C follows the trend seen for annexin V-positive cells in panel B.

To gain insight into the mechanisms of drug action against *C. neoformans*, we utilized imaging flow cytometry in combination with fluorescence detection of DM262 as well as immunostaining with a capsule-specific monoclonal antibody (MAb) detected via an Alexa Fluor 488 secondary antibody (Fig. 4 and S3). We determined through an emission and excitation scan the optimal conditions for DM262 detection via fluorescence using a range of concentrations. The optimal excitation of DM262 was achieved at 360 nm, and the maximum emission wavelength was determined to be at 396 nm (Fig. 5A and B). We were able to detect a dose-dependent fluorescence signal of DM262 within *C. neoformans* that localized to the nuclei using the 405-nm laser of the Amnis ImageStream flow cytometer. We were unable to wash out DM262 from the cells once these were incubated with the drug. DM262-treated cryptococcal cells in the range from 50 µM to 1.56 µM had fluorescence in the nuclei using the 405-nm laser while cells treated with the DMSO carrier had no comparable fluorescence (Fig. 4). Interestingly, the intensity of 18B7 staining (green in Fig. 4A and B) is highest in the 0.78 μM DM262-treated sample. This observation coincides with that in Fig. 1C where DM262 is fungistatic at 0.78 μM and fungicidal at 1.56 μM. One possible explanation is that DM262 at 0.78 μM has increased capsule synthesis as a drug-induced stress response, though the concentration is not high enough to be fungicidal. The dose-dependent increase in DM262 fluorescence was observed using either the ImageStream analysis or a SpectraMax IX3 plate reader using optimal excitation and emission wavelengths (Fig. 5B). Treatment of *C. neoformans* with DM262 had no effect on the average size of the cells (Fig. 6A). To determine if the cells were stressed by DM262, we determined the level of annexin V on the surface of the cells as a potential factor related to apoptosis-like activity of the cells (Fig. 6B, S5, and S6). A positive dose-dependent correlation between DM262 concentration and increased numbers of cells expressing annexin V was detected. Interestingly, when examining the diameter of the cells in the bright-field image compared to the 18B7 MAb-stained image, a dose-dependent difference was observed (Fig. 6C). The thickness of the capsule, calculated by the size difference in diameter between 18B7 MAb image and bright-field image and then divided by 2, showed no changes upon drug treatment. However, the maximum diameter of the cells in the 18B7 MAb-stained image varied from the DMSO control to 50 µM DM262 treatment in a dose-dependent manner (Fig. 6C). The graph shows the size difference between the longest 18B7 MAb radius and the mean bright-field radius of the cell. The trend of larger cells staining for 18B7 follows the annexin V graph very closely and is suggestive of a correlation with apoptosis-like cell death. It is, however, unclear why the bright-field image does not show a significant dose-dependent change in size of the cells. Further studies will be required to identify the potential targets of DM262.

10.1128/mSphere.00537-17.3 FIG S3  Selected plots from the ImageStream analysis of all samples. The DMSO control and 50 µM DM262 are highlighted by a different color scheme from the remaining samples. A dose-dependent increase in apoptosis-like cellular activity is visible in the first plot when viewed from low to high concentrations of DM262. Similarly, the events in the region outlined in red are increasing in a dose-dependent manner. Download FIG S3, PDF file, 0.2 MB. Copyright © 2018 Jung et al. 2018 Jung et al. This content is distributed under the terms of the Creative Commons Attribution 4.0 International license.

10.1128/mSphere.00537-17.4 FIG S4  Ten representative cells that were treated with 25 µM DM262 or the DMSO vehicle control. Each cell is individually labeled with its event number in the upper right corner, and every row represents one particular cell. A mouse monoclonal antibody, 18B7, was used to stain for the capsule of *C. neoformans*; it was visualized with an anti-mouse AF488 antibody. DM262 was visualized through fluorescence of the small molecule at 405 nm, while DRAQ5 was used as a nuclear stain in red. Cells treated with DMSO vehicle control were used as compensation matrix to account for autofluorescence of the cells. Overlays of the different channels are shown in the subsequent columns of the panel. It is obvious that no fluorescence is visible in the DM262 channel of the DMSO vehicle control. The overlay of DM262 with the nuclear stain suggests a nuclear localization of the drug. However, approximately 30% of the single cells show nuclear localization of DM262. Download FIG S4, PDF file, 0.3 MB. Copyright © 2018 Jung et al. 2018 Jung et al. This content is distributed under the terms of the Creative Commons Attribution 4.0 International license.

10.1128/mSphere.00537-17.5 FIG S5  Gating strategy of DM262-exposed cells. Cells were separated by gradient root mean square (RMS) of the bright field to identify focused cells, which were then analyzed by their aspect ratio and area in the bright field to separate single cells from multiple cells. Single cells were then analyzed for annexin V staining and DM262 fluorescence. A similar gating strategy was applied for the MAb 18B7- and DRAQ5-stained samples. Download FIG S5, PDF file, 0.5 MB. Copyright © 2018 Jung et al. 2018 Jung et al. This content is distributed under the terms of the Creative Commons Attribution 4.0 International license.

10.1128/mSphere.00537-17.6 FIG S6  Assessment of annexin V- and DM262-positive *C. neoformans* cells using ImageStream analysis. Cells were incubated at the respective DM262 concentration or DMSO control in rich medium. From each sample, 50,000 events were collected. About 50% of the cells were considered to be well in focus based on gradient RMS analysis of the bright field for further evaluation. The focused cells were then further separated by aspect ratio and area in the bright-field channel, allowing the identification of single versus multiple cells. All further analyses were then carried out on the single-cell population. Except for the sample with the highest concentration of DM262, all samples had at this step more than 23,000 single cells. The 50 µM DM262 sample had only 3,000 single cells that could be assessed. The graphs on the left of each DM262 concentration or control represent the fluorescence intensity of DM262 on the *x* axis and annexin V on the *y* axis. The orange area represents cells that were considered to be staining positively for annexin V; these values were used to generate Fig. 6B. The graph on the right shows a frequency distribution on the *y* axis versus the fluorescence intensity of DM262 on the *x* axis. The cutoff for DM262 negativity was chosen based on the DMSO control sample. A visible, concentration-dependent shift in DM262 intensity is observed in the right graph with increased DM262 concentration. Download FIG S6, PDF file, 0.1 MB. Copyright © 2018 Jung et al. 2018 Jung et al. This content is distributed under the terms of the Creative Commons Attribution 4.0 International license.

## DISCUSSION

The Malaria Box is distributed by the Medicines for Malaria Venture with the hope of identifying novel uses for drugs that have previously been identified as candidate therapies. We note that one prior study by Mayer and Kronstad also used compounds from the same organization, but the drugs tested were from the Pathogen Box, which had no compounds in common with the Malaria Box in this study (19). It is worth noting that the compound discovered by Mayer and Kronstad was active only in minimal medium, while when it was tested in rich medium no inhibitory effect of the compound on fungal cell growth was observed. In contrast, DM262 was tested in rich medium and showed dramatic antifungal activity toward multiple fungal pathogens. We have shown that DM262 has significant antifungal activity against two common fungal pathogens, *C. neoformans* and *C. albicans*, as well as against two less common fungal pathogens, *Lomentospora prolificans* and *Cryptococcus gattii*. While DM262 efficiency against *C. gattii* appears to be comparable at 72 h of treatment to that of fluconazole (Table 2), the growth kinetics of this fungus in the presence of DM262 revealed a significant growth delay. Cells grown with DM262 at 12.5 μM did not reach log phase until approximately 48 h, while the same concentration of fluconazole caused only a slight shift in log-phase growth (Fig. 2E and F, gray lines). A combination therapy approach with DM262 might lead to a more successful antifungal activity since a different molecular target is likely to be engaged in its inhibitory activity. While the specific target of DM262 is unknown, it is worth noting that a strong correlation between our growth assays and the amount of annexin V-positive cells via ImageStream analysis is observed in a dose-dependent manner, suggesting that the cells indeed undergo an apoptosis-like cell death process upon treatment with DM262 in the low-micromolar range.

After identifying DM262 as the most potent antifungal compound against four unique fungal species, we investigated the toxicity in mammalian cells. Prior studies by Van Voorhis et al. have shown a DM262 (MMV665943) 50% inhibitory concentration (IC_50_) value of 11.4 μM against MRC-5 human fibroblast cells (18). To assess the toxicity in primary murine bone marrow-derived macrophages and J774 murine macrophage-like immortalized cell lines, we examined cytotoxicity and metabolic activity with lactate dehydrogenase (LDH) and MTT assays, respectively. We found an LD_50_ of approximately 12.5 μM DM262, aligning with the previous studies in MRC-5 human fibroblasts by Van Voorhis et al. (18).

There is some potential room for improvement of this compound by chemically modifying the unreactive aniline groups of DM262 for increased solubility, which would improve delivery since DM262 was insoluble in water, phosphate-buffered saline (PBS), or ethanol but soluble to 100 mM in 100% DMSO (data not shown). In our study, DM262 was prepared in 100% DMSO and applied to fungal and mammalian cells in 2-fold serial dilutions. The final DMSO concentration in the bioassays was kept at 0.5%, adhering to the widely accepted DMSO concentration in metabolic assays. While the oral bioavailability is currently unknown for DM262, intravenous systemic delivery is still potentially viable despite its solubility profile.

It is worth noting that we observed a strong dose-dependent correlation between the amount of inhibition in our growth assays and annexin V staining with ImageStream analysis, suggesting that the cells indeed undergo apoptosis-like cell death upon treatment with DM262 in the low-micromolar range.

The observed increase of the capsular polysaccharide may suggest a defense mechanism of the fungal pathogen to decrease the inhibitory effect of DM262. Future experiments with low-dose DM262 treatment over a long period of time to establish a resistant strain may provide clues toward upregulation of defense mechanisms. In this case, DM262 may serve as a chemical probe to identify and investigate novel pathways. It will be interesting to learn if the four tested fungal species adapt in a similar way to low-dose treatment; if so, then a common target could provide additional avenues that are worth pursuing for a target-directed drug discovery approach.

Our drug-free recovery experiments show that cells exposed to DM262 did not recover on rich medium at concentrations of 1.56 µM and higher. This concentration of compound also coincided with the increase of annexin V in the culture, as we noticed an increase in annexin V-positive cells at 1.56 μM. Interestingly, at 0.78 μM DM262, the highest concentration that allowed for cellular recovery, there was a marked increase in 18B7 staining, implying increased capsule synthesis. At 0.78 μM DM262, there may be a drug-induced stress response to generate capsular polysaccharide where DM262 is stressing the cryptococcal cultures without being fungicidal. At 1.56 μM, there is approximately a 7-fold increase of annexin V compared to the DMSO control. At this concentration, the cells are already nonviable after transfer to drug-free rich medium plates. At 3.13 µM, the next higher concentration tested, the levels of annexin V compared to the control peak at about 40-fold. Even after multiple days of incubation on rich medium plates, no viable cells were observed.

In summary, we have identified a potential new antifungal drug, DM262, which is able to inhibit and delay growth at submicromolar concentrations and exhibits fungicidal activity at a greater than 1.56 µM concentration in the best possible medium for optimal fungal growth. We anticipate that our drug, when tested in minimal medium, would show a fungicidal effect at even lower drug concentrations. The scaffold structure is novel among drugs commonly used to treat fungal diseases, implying the possibility of a new class of drugs. The benzimidazole scaffold is easily modified and tractable through synthesis, making it attractive for potential modifications. We have tested compounds similar to DM262 with modified side groups that were commercially available, though antifungal activity was weaker than that of DM262 (data not shown). This shows that the efficacy of the drug can be modified, and future work may provide a derivative with higher antifungal activity and fewer off-target effects. The next step in the development of this novel drug is to identify its target pathway. The ability to visualize DM262 fluorescence will allow us to more easily identify a potential target pathway. With a target identified, we may be able to modify the compound to increase specificity to decrease toxicity and off-target inhibition.

## MATERIALS AND METHODS

### Strains.

H99 (serotype A) is a well-characterized wild-type laboratory reference strain of *Cryptococcus neoformans* (20). H99 was selected because it is a virulent strain widely used by the field. *Cryptococcus gattii* strain R265 is used as the primary reference strain on which the genome is based (ATCC MYA-4093). This strain was isolated during the Vancouver Island (British Columbia, Canada) outbreak of 2004 (21, 22). H99 and R265 were maintained in Sabouraud (Sab) dextrose broth at 30°C with constant 200-rpm agitation. One day prior to each experimental drug treatment, Sab broth was inoculated from glycerol stock and cells were grown overnight to log phase before washing with PBS and resuspending in fresh Sab broth prior to treatment.

The *Lomentospora prolificans* strain used in this study is a naturally antifungal-resistant clinical isolate from an 11-year-old boy in Australia (23) (*L. prolificans* AMMRL 140.04, catalogue no. 90853; ATCC, Manassas, VA). AMMRL 140.04 was grown on Sab dextrose rich medium plates and incubated for 5 to 7 days at 30°C prior to rinsing the surface with PBS to collect conidia for drug treatment dilution series assays.

*Candida albicans* strain SC5314 is one of the original background strains used in systematic sequencing of the genome (24). SC5314 is a common laboratory strain that is virulent in mouse models. *C. albicans* was streaked from frozen glycerol stocks on Sab rich medium plates before inoculation in Sab dextrose broth prior to overnight growth at 30°C with constant 200-rpm agitation.

### Compound library.

A collection of 400 compounds was made available by the Medicines for Malaria Venture (Geneva, Switzerland) in the form of the open-access MMV Malaria Box (16). Compounds were aliquoted to prevent freeze-thaw cycles and stored at −80°C in 100% DMSO at a concentration of 10 mM. Before usage, compounds were thawed at room temperature and thoroughly mixed. Compounds were diluted in a 2-fold series from 10 mM down to 0.8 mM with DMSO before subsequent growth inhibition assays.

### Growth inhibition assays.

The effectiveness of these MMV Malaria Box compounds against *C. neoformans* was inferred by their ability to inhibit growth, as measured by turbidity over time. The Bioscreen C instrument (Growth Curves USA, Piscataway, NJ) allows us to analyze ~60 compounds at a time in triplicate with constant temperature and agitation. Turbidity was measured at 420 to 580 nm (wideband range) every 15 min for 72 h at 37°C to simulate physiologically relevant temperatures. According to the manufacturer’s protocol, wideband is generally used for turbidity measurements, as it is mostly insensitive to changes in color. All compounds were suspended in dimethyl sulfoxide (DMSO), and each test run of 60 compounds had an independent set of DMSO vehicle and medium-alone controls. We inoculated 5 × 10^3^ cells of *C. neoformans* H99 and *C. albicans* SC5314 in 200 μl of Sabouraud dextrose broth in each of the honeycomb (HC-2) plate’s 100 wells. A 2-fold dilution series of each compound from 50 μM down to a 0.39 μM final concentration were tested for effects against *C. neoformans*. To differentiate fungistatic and fungicidal activity, 5 μl of each well after 72 h was spotted on Sabouraud dextrose agar rich medium plates and allowed to grow at the optimal temperature of 30°C to induce a full recovery of viable yeast. Colony spots were noted after 24 h to identify fungicidal versus fungistatic concentrations of each compound. We examined the efficiency of the top five compounds against *C. neoformans* and *C. albicans*.

### Mammalian cell viability and toxicity assays.

Bone marrow was isolated from 7- to 14-week-old C57BL/6 mice and differentiated at 37°C and 9.5% CO_2_ for 7 days with bone marrow-derived macrophage (BMDM) medium consisting of Dulbecco modified Eagle medium (DMEM), 20% L929 conditioned medium, 10% fetal bovine serum (FBS), 1% nonessential amino acids, 1% Gibco GlutaMAX (Thermo Fisher Scientific, Halethorpe, MD), 1% HEPES, 1% penicillin-streptomycin (Pen-Strep), and 0.1% β-mercaptoethanol. BMDM (5 × 10^4^) were plated in 200 μl/well in a 96-well format. For the MTT colorimetric assay, thiazolyl blue tetrazolium bromide (Sigma; catalogue no. M2128) was added to cells where it is reduced into pigmented formazan crystals by viable mammalian cells. Cells were incubated for 2 h to metabolize the tetrazolium salts before lysing with extraction buffer (12.5% SDS and 45% dimethylformamide [DMF]) overnight. The following day, absorbance was measured at 570 nm in an E_Max_ Plus microplate reader (Molecular Devices, CA, USA).

In addition to MTT measurements, we also measured lactate dehydrogenase (LDH), a cytoplasmic enzyme that is released when cell lyse and die. The CytoTox 96 nonradioactive cytotoxicity assay (Promega; catalogue no. G1781) was used to measure the lytic release of LDH. After a 2-h exposure of compound, the supernatant was removed and incubated with substrate, and assay buffer was added for 30 min before acetic acid was added as a stop solution. Absorbance was measured at 570 nm in an E_Max_ Plus microplate reader.

### Fluorescence of DM262.

We assessed the fluorescence of 1 M DM262 and DMSO vehicle control alone, using the Fluorilog-3 spectrophotometer (Horiba Jobin Yvon, Edison, NJ). To identify the optimal excitation wavelength, we did a broad-spectrum excitation scan of every odd wavelength between 300 and 500 nm, which showed 391 to 393 nm as the optimum excitation wavelength (see Fig. S1 in the supplemental material). Subsequently, we excited at 340 nm and scanned between 355 and 500 nm for optimal emission wavelengths.

### Imaging flow cytometry on *C. neoformans*.

*C. neoformans* was grown in rich liquid Sabouraud medium (200 rpm, 30°C) in the presence of DM262 ranging from 50 µM to 0.39 µM with a final DMSO concentration of 0.5% in the culture as well as a control culture with 0.5% DMSO for 24 h. Cells were harvested and washed twice with ice-cold 1× PBS to remove medium components as well as any DM262 remaining in the culture medium. Samples for Amnis ImageStream^X^ MK II (Amnis Corporation, Seattle, WA) analysis were fixed with freshly prepared 4% paraformaldehyde in 1× PBS for 40 min under constant rotation at room temperature. Each sample was split into two parts, and one was stained with 18B7, a monoclonal antibody specific for the polysaccharide capsule (25), while DRAQ5 was utilized for the nuclear stain. The second sample was kept unstained to be able to detect cellular autofluorescence as well as fluorescence of DM262. The untreated DMSO vehicle control was used to generate a compensation matrix correcting for the intrinsic autofluorescence of the cells. Events were counted based on the bright-field channel cell area being greater than 12 to include all cells (single cells and clumps). The instrument was set to a magnification of ×60 with extended depth of field (EDF) turned on and all lasers at their maximum power except for the side-scatter laser (405 nm, 90 mW; 488 nm, 100 mW; 658 nm, 120 mW; 768 nm, 3.25 mW), and 25,000 events were collected per sample. Analysis of the data was carried out with Ideas 6.2 (Amnis Corporation, Seattle, WA) with the following gating strategy. First, the cells were separated based on their contrast in the bright field to determine focused cells. Second, focused cells were separated by their aspect ratio and total bright-field area to identify single versus multiple cells. The single cells were then used for detailed analysis of DNA content, amount of annexin V, and/or DM262 fluorescence as well as specific capsule staining with 18B7. In a separate set of experiments, *C. neoformans* was exposed to DMSO vehicle control or DM262 ranging from 50 µM to 0.39 µM for 24 h, and the amount of annexin V as a marker for apoptosis-like cell death was assessed.
