# Supplementary figures and images for: Novel Antifungal Compounds Discovered in Medicines for Malaria Venture’s Malaria Box
Source: mSphere. 2018 Mar 14;3(2):e00537-17. doi: 10.1128/mSphere.00537-17 (PMC5853493; doi:10.1128/mSphere.00537-17)

Supplemental Figure S1

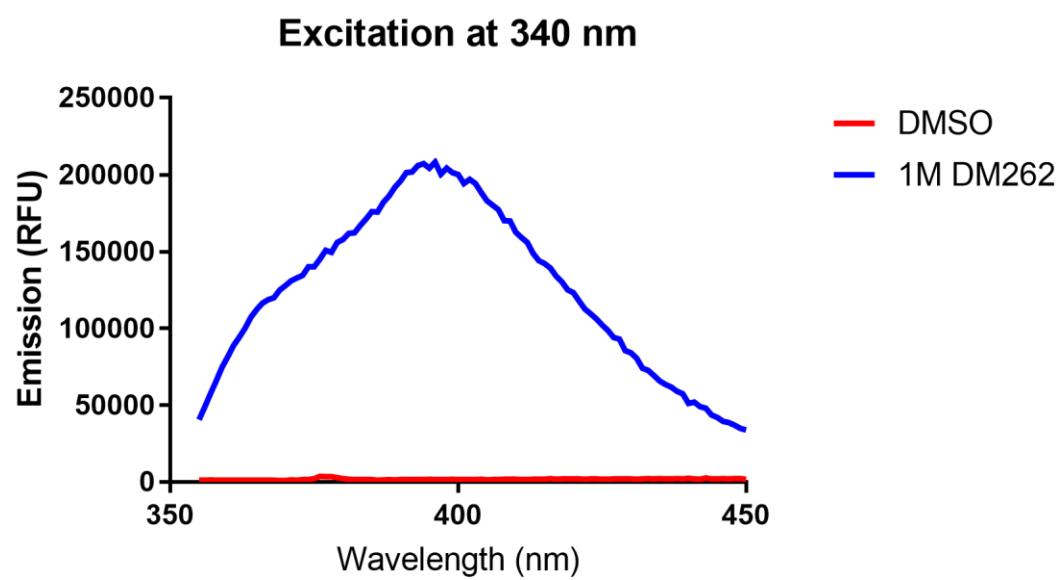

Supplement: FIG S1 [file sph001182485sf1.pdf]

## Supplemental Figure S2

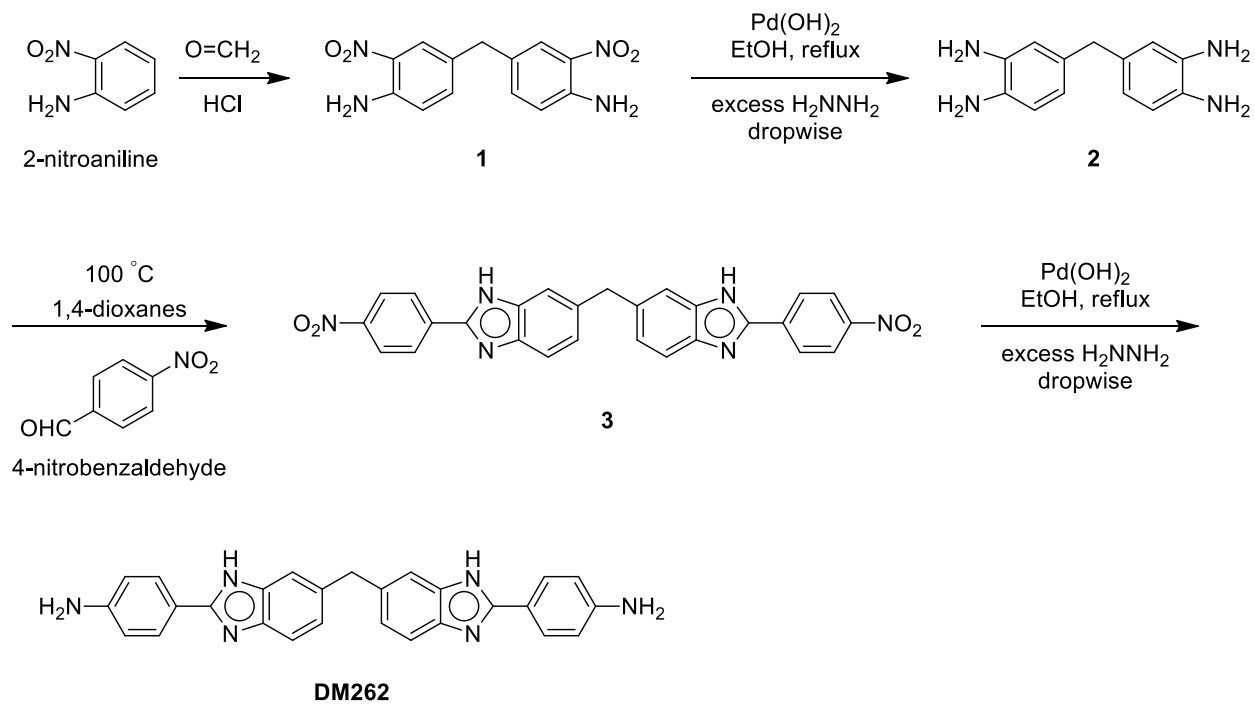

Supplement: FIG S2 [file sph001182485sf2.pdf]

Supplemental Figure S3

ImageStream detailed analysis of DM262 dose dependency:

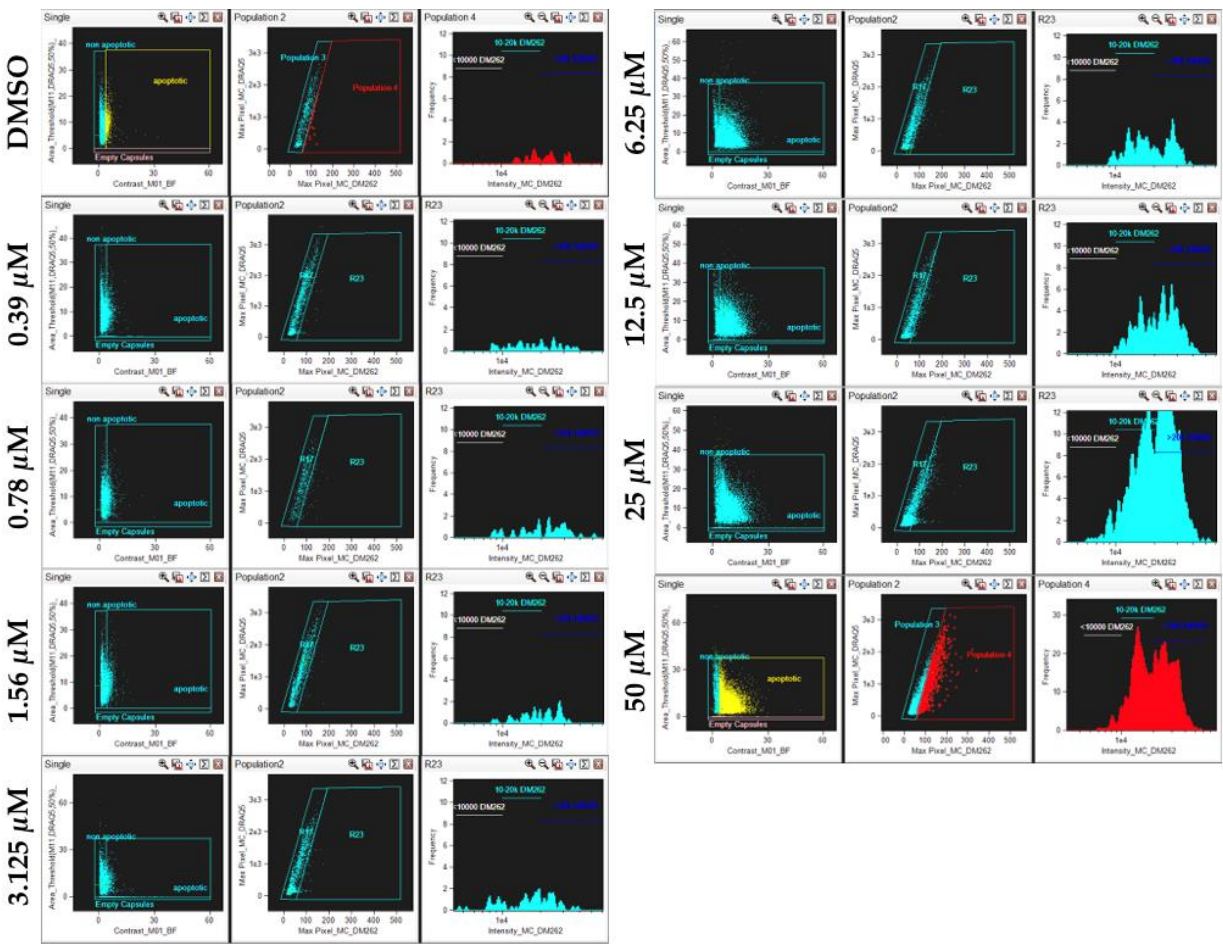

Supplement: FIG S3 [file sph001182485sf3.pdf]

25 $\mu$ M DM262

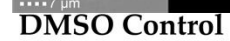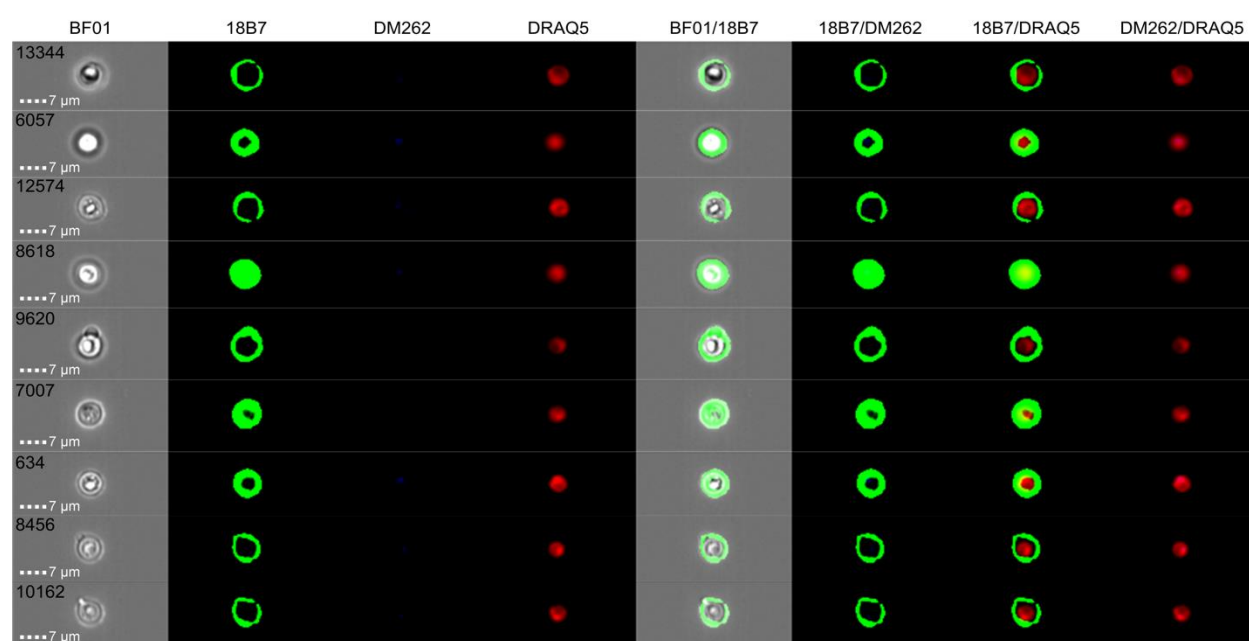

Supplement: FIG S4 [file sph001182485sf4.pdf]

Supplemental Figure S5

DMSO Control

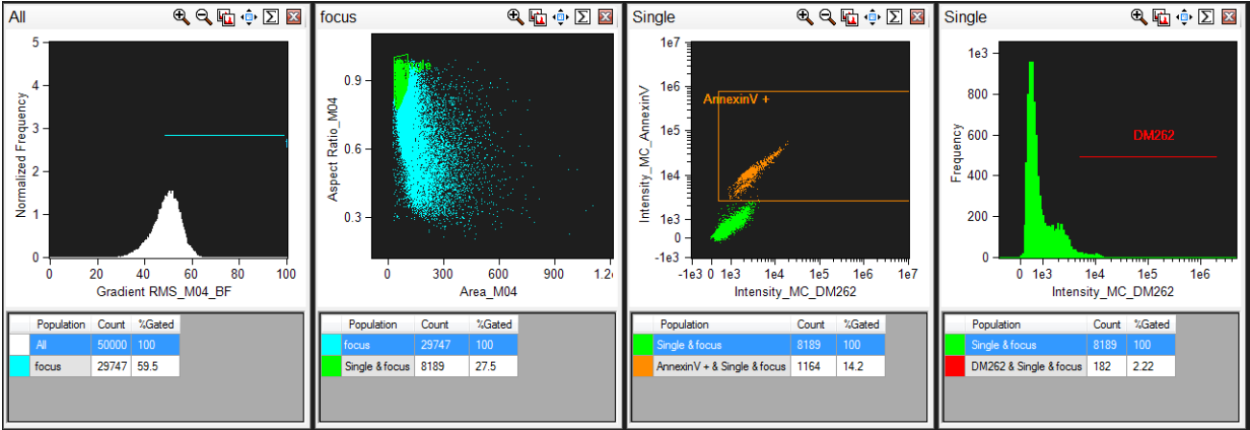

DM262 12.5  $\mu$ M

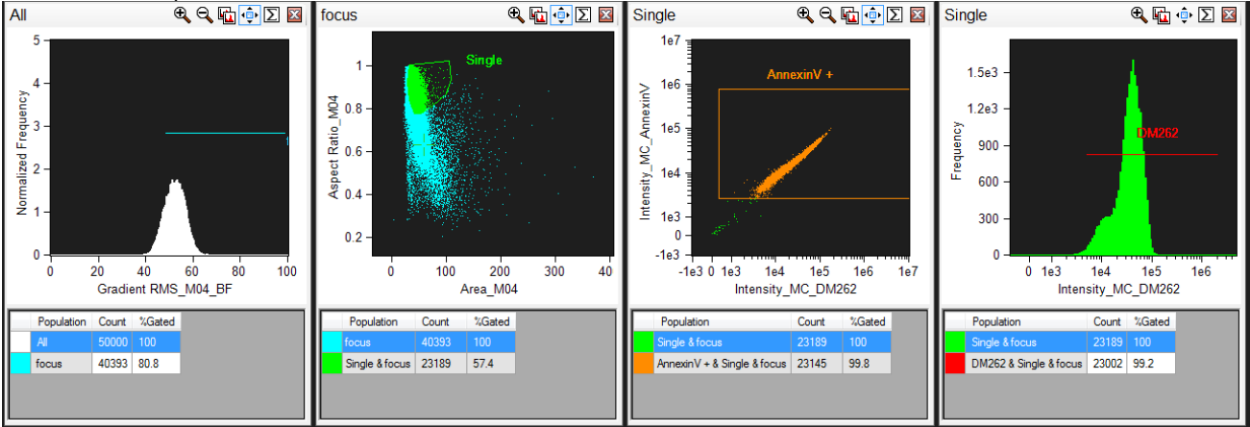

DM262 50  $\mu$ M

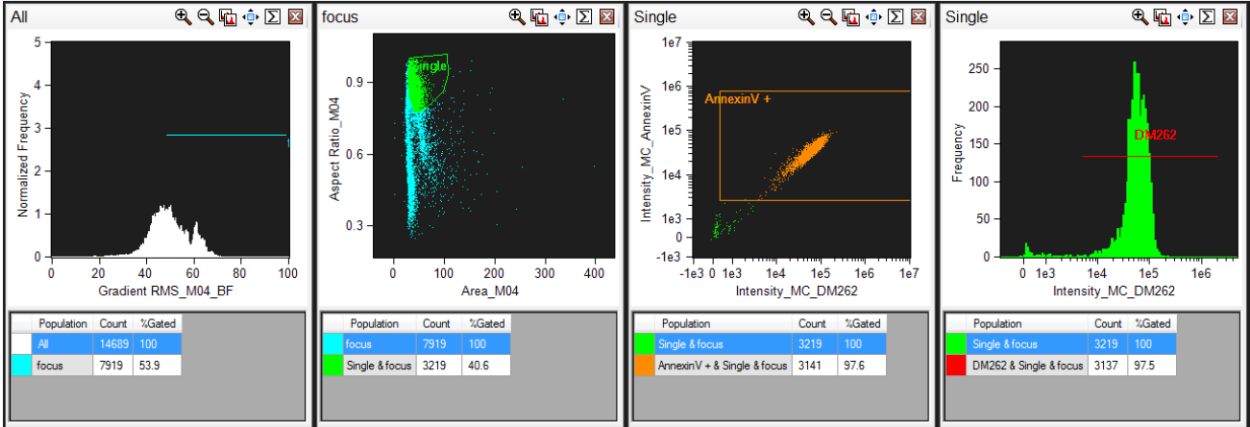

Supplement: FIG S5 [file sph001182485sf5.pdf]

Supplemental Figure S6

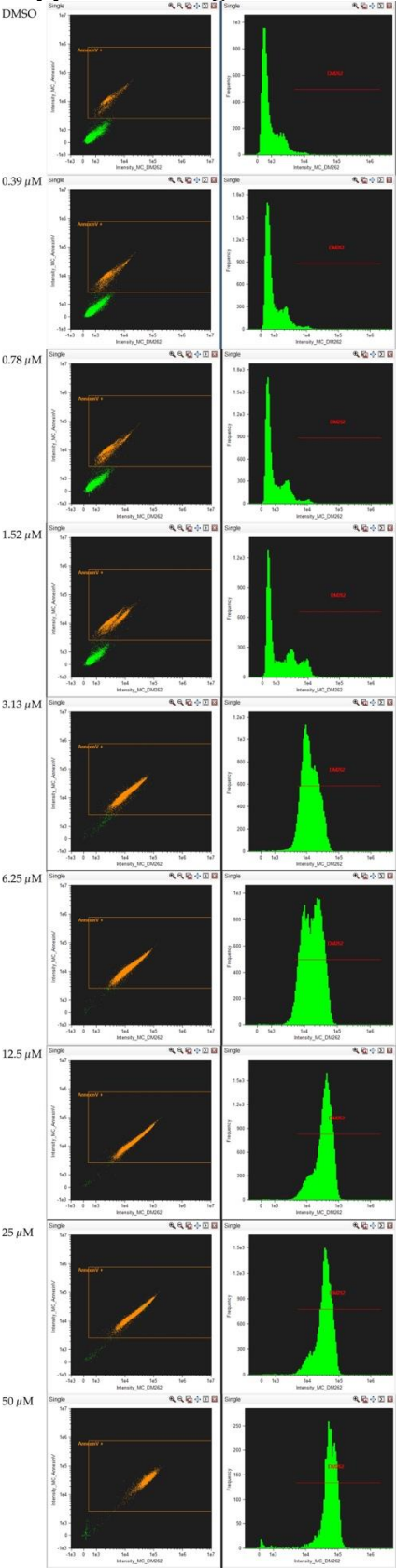

Supplement: FIG S6 [file sph001182485sf6.pdf]
